# Multiple organ infarction caused by aortic thrombus in a lung cancer patient with the *BRAF* mutation

**DOI:** 10.1016/j.rmcr.2022.101608

**Published:** 2022-02-18

**Authors:** Hirofumi Watanabe, Masato Karayama, Yusuke Inoue, Hironao Hozumi, Yuzo Suzuki, Kazuki Furuhashi, Tomoyuki Fujisawa, Noriyuki Enomoto, Yutaro Nakamura, Naoki Inui, Takafumi Suda

**Affiliations:** a Second Division, Department of Internal Medicine, Hamamatsu University School of Medicine, 1-20-1 Handayama, Hamamatsu, 431-3192, Japan; b Department of Chemotherapy, Hamamatsu University School of Medicine, 1-20-1 Handayama, Hamamatsu, 431-3192, Japan; c Department of Clinical Pharmacology and Therapeutics, Hamamatsu University School of Medicine, 1-20-1 Handayama, Hamamatsu, 431-3192, Japan

**Keywords:** Arterial thromboembolism, BRAF inhibitor, MEK inhibitor, Oncogene

## Abstract

A 72-year-old male patient with advanced lung adenocarcinoma harboring a *BRAF* mutation had received treatment with a BRAF inhibitor and a MEK inhibitor. Treatment was ceased after 40 days because of disease progression. Twenty-four days after treatment cessation, the man was referred to our hospital with worsening abdominal and back pain over 2 weeks. Computed tomography revealed a massive thrombus in the descending aorta, bilateral kidney infarction, splenic infarction, and intestinal enlargement due to ileus. He was diagnosed with multiple organ infarction caused by arterial thromboembolism. Tumors harboring *BRAF* mutations and BRAF/MEK inhibitor therapy both have the potential to increase thrombosis risk, and were therefore thought to be associated with the occurrence of aortic thrombosis.

## 1 Introduction

Aortic thrombosis is a rare disease that often presents with fatal thromboembolism such as cerebral infarction, myocardial infarction, and acute limb ischemia [[1], [2], [3]]. Pathogenic mechanisms of aortic thrombosis include hypercoagulability, endothelial injury, or morphological abnormalities of the aorta [[1], [2], [3], [4]]. Generally, aortic thrombosis occurs in association with systemic diseases such as cancer, infection, coagulation disorders, or vasculitis. Other known risk factors for aortic thrombosis include chest trauma, smoking, hypertension, atherosclerosis and drugs that induce endothelial injury and/or enhance platelet activation [[4], [5], [6]].

Cancer is a well-known risk factor for arterial thrombosis. In a large cohort study of 279,719 patients with various cancers, the 6-month cumulative incidence of arterial thrombosis was reported to be 4.7%, but was 2.2% in matched control patients [7]. Patients with lung cancer in particular had the highest rates of arterial thrombosis (8.3%). Furthermore, oncogenic driver genes are reported to be associated with thrombosis [8]. The V-Raf murine sarcoma viral oncogene homolog B1 (*BRAF*) mutation acts as an oncogenic driver gene in solid cancers including lung cancer [[9], [10], [11]]. *BRAF*-mutated tumors have the potential to increase thrombosis risk, compared with non- *BRAF*-mutated tumors [12,13]**.**

Meanwhile, thrombosis is also known as an adverse event of cancer therapy. Combination therapy with a BRAF inhibitor and a mitogen-activated protein kinase kinase (MEK) inhibitor is the standard treatment for solid cancers harboring *BRAF* mutations [[9], [10], [11]]. Combination therapy with BRAF/MEK inhibitors have the potential to be associated with vasospasm, platelet activation, and hypercoagulation [14,15]. Thrombosis is a rare adverse event. However, the association between BRAF/MEK inhibitor therapy and an increased risk of thromboembolism has been reported [14,16].

We herein present a case of multiple organ infarction caused by massive thrombus in descending aorta in a patient who received a BRAF inhibitor (dabrafenib) and a MEK inhibitor (trametinib) for the treatment of advanced lung adenocarcinoma harboring the *BRAF* V600E mutation.

## 2 Case presentation

**Fig. 1:**
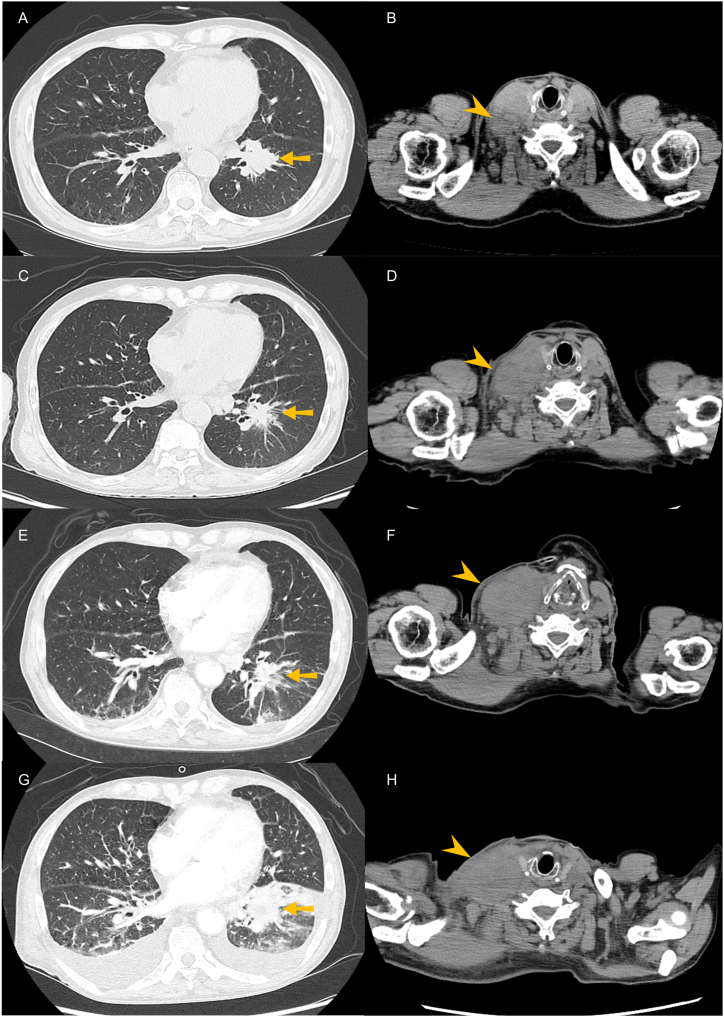
Computed tomography images of lung cancer. Computed tomography revealed the primary tumor in the left lower lobe of the lungs (arrows) and right cervical lymph node metastasis (arrowheads) at diagnosis (A, B). Following treatment with dabrafenib and trametinib, the primary tumor shrank, whereas the cervical lymph node metastasis was enlarged (C, D). At admission because of multiple organ infarction, the primary tumor remained controlled, but the cervical lymph node metastasis had progressed (E, F). Despite second-line treatment with pembrolizumab, the disease further progressed (G, H).

A 72-year-old male was referred to our hospital with right cervical lymph node swelling. His medical history included hypertension, hyperlipidemia, and chronic obstructive pulmonary disease. He had a 45-pack-year history of smoking before the diagnosis of lung cancer. Transbronchial biopsy from the primary tumor of the left lower lobe of the lungs revealed lung adenocarcinoma. He was diagnosed as having advanced lung adenocarcinoma, with adrenal, bone, and cervical lymph node metastases (cT2aN3M1c, stage IV, Fig. 1A and B). Next-generation sequencing using the transbronchial biopsy tissue revealed a *BRAF* V600E mutation without any other concomitant oncogenes (Oncomine Dx Target Test ®, Thermo Fisher Scientific, Waltham, MA, USA). The tumor proportion score of programmed death ligand-1 was 10% (Dako 22C3 pharmDx, Agilent Technologies, Santa Clara, CA, USA). He had received dabrafenib and trametinib as first-line treatment. From 12 days after the start of the treatment, fever of unknown origin occurred and persisted despite 10 days of antibiotic treatment with levofloxacin. Following treatment with dabrafenib and trametinib, the primary tumor in the left lower lobe of the lungs shrank, but the cervical lymph node metastasis was enlarged (Fig. 1C and D). Therefore, dabrafenib and trametinib were ceased 40 days after the start of treatment. Fever of unknown origin continued even after the cessation of dabrafenib and trametinib. Twenty-four days after the cessation of the treatment, he was admitted to our hospital with gradually worsening abdominal pain and back pain over 2 weeks.

**Fig. 2:**
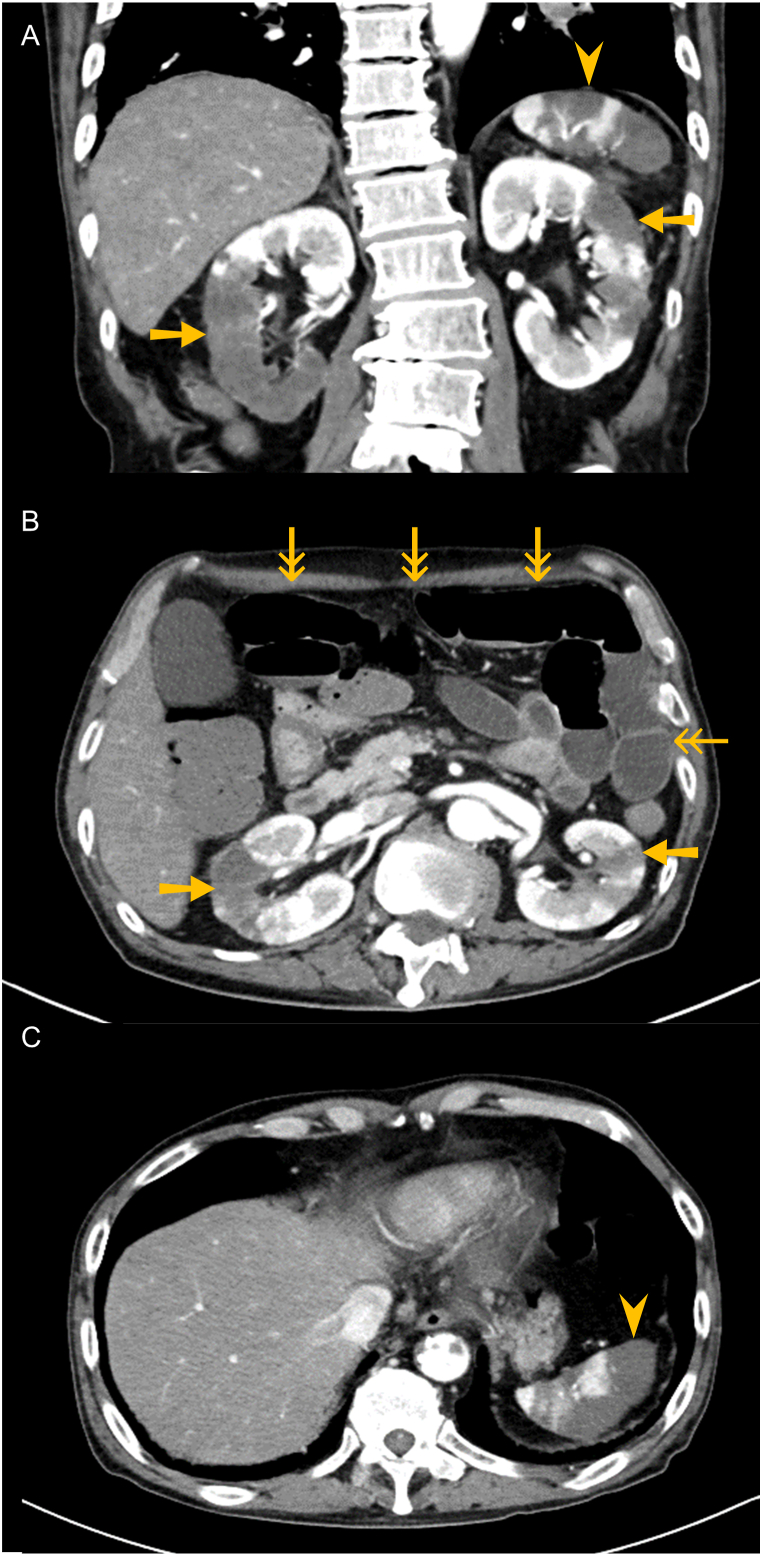
Enhanced abdominal computed tomography images. Enhanced abdominal computed tomography revealed bilateral kidney infarction (arrows), splenic infarction (arrowheads), and intestinal enlargement due to ileus (double arrows) on admission. Coronal (A) and horizontal (B, C) images.

**Fig. 3:**
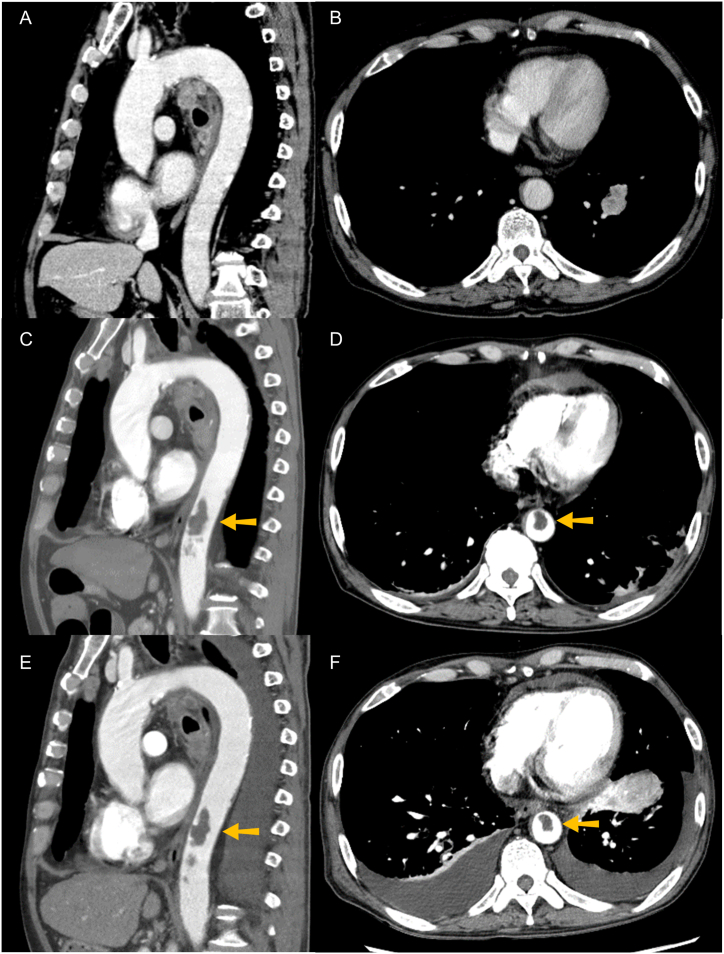
Enhanced computed tomography angiography images of aorta. There was no thrombus in the aorta at the lung cancer diagnosis (A, B). A massive thrombus was observed in the descending aorta (arrows) at the first day of admission (C, D). There was no obvious shrinkage in the aortic thrombus after 20-day anti-platelet therapy with aspirin and alprostadil (E, F).

He had a body temperature of 37.5 °C, blood pressure of 100/66 mmHg, and heart rate of 100 beats/min. Laboratory examinations revealed increased white blood cell counts (25,480/μL; 90.0% neutrophils), and increased C-reactive protein (18.4 mg/dL), lactate dehydrogenase (2705 IU/L), and D-dimer (26.5 μg/mL). (Table 1). Enhanced whole-body computed tomography revealed bilateral kidney and splenic infarction and intestinal enlargement due to ileus (Fig. 2A–C). There was a massive thrombus in the descending thoracic aorta, which had not been observed at the lung cancer diagnosis (Fig. 3A–D). He was diagnosed with multiple organ infarction caused by arterial thromboembolism. The primary tumor in the left lower lobe remained controlled, but the cervical lymph node metastasis had progressed further (Fig. 1E and F). Lupus anticoagulant, anti‐cardiolipin antibody, myeloperoxidase anti-neutrophil cytoplasmic antibody (ANCA), and proteinase 3-ANCA were not detected. Blood culture was also negative. He had normal echocardiography and no arrhythmias.

**Table 1:** Laboratory findings on admission.

WBC,/μL	25480	Total protein, g/dL	6.9	HbA1c, %	6.4
Neutrophil, %	90.0	Total bilirubin, mg/dL	0.5	Blood sugar, mg/dL	123
Lymphocyte, %	4.5	LDH, IU/L	2705	CEA, ng/mL	37.8
Monocyte, %	5.0	AST, U/L	73	CYFRA, ng/mL	70.8
Eosinophil, %	0.0	ALT, U/L	120	SLX, U/mL	36
Basophil, %	0.5	Albumin, g/dL	2.2	PR3-ANCA, U/mL	<1.0
RBC, ×10⁴/μL	389	CRP, mg/dL	18.4	MPO-ANCA, U/mL	<1.0
Hemoglobin, g/dL	10.8	Amylase, U/L	83	Anti-nuclear antibody	<1:40
PLT, ×10⁴/μL	34.2	D-dimer, μg/mL	26.5	Anti-cardiolipin antibody, U/mL	<1.2
BUN, mg/dL	21.0	Fibrinogen, mg/dL	558	Lupus anticoagulant, N. R	<1.2
Creatinine, mg/dL	0.83	PT, sec	14.3	Urinary protein	1+
Na, mmol/L	130	PT-INR	1.17	Urinary occult blood	±
K, mmol/L	3.6	APTT, sec	32.5	Urinary sugar	–
Cl, mmol/L	88	APTT, %	78	Urinary bilirubin	–

WBC: white blood cells, RBC: red blood cells, PLT: platelet, BUN: blood urea nitrogen, LDH: lactate dehydrogenase, AST: aspartate aminotransferase, ALT: alanine aminotransferase, AST: aspartate aminotransferase, ALT: alanine aminotransferase, CRP: C-reactive protein, PT: prothrombin time, PT-INR: prothrombin time-international normalized ratio, APTT: activated partial thromboplastin time, HbA1c: hemoglobin A1c, CEA: carcinoembryonic antigen, CYFRA: cytokeratin subunit 19 fragment, SLX: sialyl Lewis X-i antigen, PR3-ANCA: proteinase3 antineutrophil cytoplasmic antibody, MPO-ANCA: myeloperoxidase anti-neutrophil cytoplasmic antibody, N. R; normalized ratio.

**Fig. 4:**
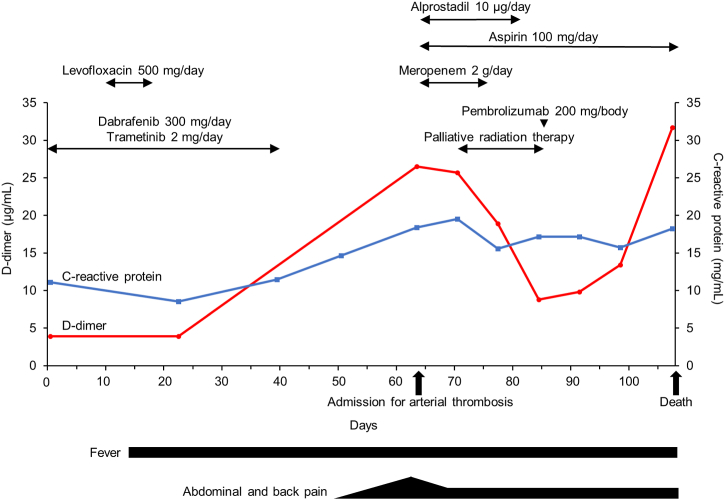
Clinical course of the patient. Blue and red lines indicated levels of C-reactive protein and D-dimer, respectively. (For interpretation of the references to color in this figure legend, the reader is referred to the Web version of this article.)

Anti-platelet therapy with aspirin and alprostadil (a prostaglandin E1 agent) was started for the aortic thrombosis. Although there was no obvious shrinkage in the aortic thrombus after the start of the anti-platelet therapy (Fig. 3E and F), the D-dimer level gradually decreased, and ileus was improved (Fig. 4). During anti-platelet therapy, the tumor progressed, and he received palliative radiation therapy for the painful cervical lymph node metastasis (a total dose of 30 Gy). His general condition gradually deteriorated to Eastern Cooperative Oncology Group performance status 2 due to cancer progression. He was expected to be unable to tolerate cytotoxic chemotherapy and therefore received pembrolizumab as second-line therapy at 24 days after the admission. However, the treatment was unsuccessful (Fig. 1G and H), and he died from the lung cancer progression at 45 days after admission (Fig. 4).

## 3 Discussion

Aortic thrombosis is associated with certain specific causes and is rarely idiopathic. In the current case, three possible factors were attributed to the occurrence of aortic thrombosis.

First, cancer is a risk factor for thrombosis. The underlying mechanisms are thought to be a hypercoagulable state induced by circulating microparticles, secretion of procoagulant factors, and increased platelet activity [17]. Furthermore, *BRAF*-mutated tumors have the potential to increase thrombosis risk. For example, Chang et al. reported that 52.9% (9/17) of patients with colorectal cancer harboring the *BRAF* mutation developed thrombosis, which was significantly more than in those without *BRAF* mutations (22.4%; 86/383) [12]. Ortega et al. reported that 28.6% (6/21) of patients with colorectal cancer harboring *BRAF* mutations developed thrombosis, compared with 21.9% (23/105) of those without *BRAF* mutation [13]**.** Although the underlying mechanisms are unknown, the activation of oncogenes induces the expression of genes associated with tissue factors that control hemostasis [8,12,13,17,18].

Second, BRAF/MEK inhibitor therapy has the potential to increase the risk of thrombosis. In 4659 patients with melanoma, lung cancer, and colorectal cancer who received BRAF/MEK inhibitors, 8.2% developed cardiovascular adverse events, including 1.4% with venous thromboembolism and 1.2% with pulmonary embolism [16]. This was significantly more frequent than the 6.1% of patients with cardiovascular adverse events, including 0.8% with venous thromboembolism and 0.6% with pulmonary embolism, in a cohort of 3053 patients undergoing BRAF inhibitor monotherapy [16]. Furthermore, in 2317 patients with melanoma, combination therapy with BRAF/MEK inhibitors was associated with an increased risk of pulmonary embolism compared with BRAF monotherapy (relative risk, 4.36; 95% confidence interval, 1.23–15.44) [14]. Combination therapy with BRAF/MEK inhibitors interferes with the mitogen-activated protein kinase (MAPK) cascade that is associated with the production of prostacyclin (PGI_2_) and nitric oxide (NO). Inhibition of PGI_2_ and NO leads to vasospasm, platelet activation, and hypercoagulation [14,15]. It is possible that BRAF/MEK inhibitors are associated with thrombosis via inhibition of PGI_2_ and NO.

Third, comorbidities are also risk factors for aortic thrombosis [[4], [5], [6]]. The patient in our case had a history of smoking, hypertension, and hyperlipidemia, which might have affected the development of thrombosis. In the current case, it was unclear which of the three factors had the strongest impact on the occurrence of aortic thrombosis. However, the D-dimer level, which was temporarily reduced after anti-platelet therapy, eventually increased again with the progression of the tumor. The clinical course suggests that tumor-induced hypercoagulation may have had the dominant effect on formation of the aortic thrombus in this patient, rather than it being an adverse event of the BRAF/MEK inhibitors.

## 4 Conclusion

Although aortic thrombosis is rare, patients with lung cancer with the *BRAF* V600E mutation who receive BRAF/MEK inhibitor therapy may be at risk of thrombosis. Therefore, careful attention to the possibility of thrombotic events is warranted.

## Statement confirming consent

Appropriate written informed consent was obtained for publication of this case report and accompanying images.

## Declarations of interest

None.
